# Translational randomized phase II trial of cabozantinib in combination with nivolumab in advanced, recurrent, or metastatic endometrial cancer

**DOI:** 10.1136/jitc-2021-004233

**Published:** 2022-03-14

**Authors:** Stephanie Lheureux, Daniela E Matei, Panagiotis A Konstantinopoulos, Ben X Wang, Ramy Gadalla, Matthew S Block, Andrea Jewell, Stephanie L Gaillard, Michael McHale, Carolyn McCourt, Sarah Temkin, Eugenia Girda, Floor J Backes, Theresa L Werner, Linda Duska, Siobhan Kehoe, Ilaria Colombo, Lisa Wang, Xuan Li, Rachel Wildman, Shirin Soleimani, Scott Lien, John Wright, Trevor Pugh, Pamela S Ohashi, David G Brooks, Gini F Fleming

**Affiliations:** 1 Drug Development Program, Department of Medical Oncology and Hematology, Princess Margaret Cancer Centre, Toronto, Ontario, Canada; 2 Department of Obstetrics and Gynecology, Indiana University Melvin and Bren Simon Cancer Center, Indianapolis, Illinois, USA; 3 Department of Gynecologic Oncology, Dana-Farber Cancer Institute, Boston, Massachusetts, USA; 4 Immune Profiling Team – Tumor Immunotherapy Program, Princess Margaret Cancer Centre, Toronto, Ontario, Canada; 5 Department of Medical Oncology, Mayo Clinic, Rochester, Minnesota, USA; 6 Department of Gynecologic Oncology, University of Kansas Medical Center, Kansas City, Kansas, USA; 7 Department of Gynecology and Obstetrics, Johns Hopkins School of Medicine, Baltimore, Maryland, USA; 8 Department of Obstetrics and Gynecology, Moores Cancer Centre, UC San Diego Health, La Jolla, California, USA; 9 Department of Gynecology Oncology, Washington University School of Medicine, St Louis, Missouri, USA; 10 Department of Gynecology Oncology, Virginia Commonwealth University, Richmond, Virginia, USA; 11 Department of Gynecology Oncology, Rutgers Cancer Institute of New Jersey, New Brunswick, New Jersey, USA; 12 Department of Gynecologic Oncology, Ohio State University, Columbus, Ohio, USA; 13 Division of Oncology, Department of Medicine, Huntsman Cancer Institute, University of Utah, Salt Lake City, Utah, USA; 14 Department of Gynecology Oncology, University of Virginia, Charlottesville, Virginia, USA; 15 Department of Gynecology Oncology, NYU Langone, New York City, New York, USA; 16 Department of Statistics, Princess Margaret Cancer Centre, Toronto, Ontario, Canada; 17 Department of Medical Biophysics, University of Toronto, Toronto, Ontario, Canada; 18 Cancer Genomics Program, Princess Margaret Cancer Centre, Toronto, Ontario, Canada; 19 Department of Immunology, University of Toronto, Toronto, Ontario, Canada; 20 Investigational Drug Branch, Cancer Therapy Evaluation Program, National Cancer Institute, Bethesda, Maryland, USA; 21 Department of Immunology, Princess Margaret Cancer Centre, Toronto, Ontario, Canada; 22 Department of Medicine, University of Chicago Medicine, Chicago, Illinois, USA

**Keywords:** immunotherapy, biomarkers, tumor, clinical trials, phase II as topic, drug therapy, combination, genital neoplasms, female

## Abstract

**Background:**

Combining immunotherapy and antiangiogenic agents is a promising treatment strategy in endometrial cancer. To date, no biomarkers for response have been identified and data on post-immunotherapy progression are lacking. We explored the combination of a checkpoint inhibitor (nivolumab) and an antiangiogenic agent (cabozantinib) in immunotherapy-naïve endometrial cancer and in patients whose disease progressed on previous immunotherapy with baseline biopsy for immune profiling.

**Patients and methods:**

In this phase II trial (ClinicalTrials.gov NCT03367741, registered December 11, 2017), women with recurrent endometrial cancer were randomized 2:1 to nivolumab with cabozantinib (Arm A) or nivolumab alone (Arm B). The primary endpoint was Response Evaluation Criteria in Solid Tumors-defined progression-free survival (PFS). Patients with carcinosarcoma or prior immune checkpoint inhibitor received combination treatment (Arm C). Baseline biopsy and serial peripheral blood mononuclear cell (PBMC) samples were analyzed and associations between patient outcome and immune data from cytometry by time of flight (CyTOF) and PBMCs were explored.

**Results:**

Median PFS was 5.3 (90% CI 3.5 to 9.2) months in Arm A (n=36) and 1.9 (90% CI 1.6 to 3.4) months in Arm B (n=18) (HR=0.59, 90% CI 0.35 to 0.98; log-rank p=0.09, meeting the prespecified statistical significance criteria). The most common treatment-related adverse events in Arm A were diarrhea (50%) and elevated liver enzymes (aspartate aminotransferase 47%, alanine aminotransferase 42%). In-depth baseline CyTOF analysis across treatment arms (n=40) identified 35 immune-cell subsets. Among immunotherapy-pretreated patients in Arm C, non-progressors had significantly higher proportions of activated tissue-resident (CD103+CD69+) ɣδ T cells than progressors (adjusted p=0.009).

**Conclusions:**

Adding cabozantinib to nivolumab significantly improved outcomes in heavily pretreated endometrial cancer. A subgroup of immunotherapy-pretreated patients identified by baseline immune profile and potentially benefiting from combination with antiangiogenics requires further investigation.

## Introduction

Endometrial cancer (EC) is the most common gynecologic cancer in North America, and its incidence continues to rise.^1^ Women with advanced or recurrent EC have a poor prognosis (5-year survival <20%).^2^ Initial therapy for unresectable recurrent/metastatic disease is typically carboplatin plus paclitaxel. For those with potentially endocrine‐sensitive tumors, an endocrine therapy trial can be appropriate.^1^ Response rates remain below 50% and responses are typically transient.^1^

Recent EC subclassification has led to an increasingly targeted treatment approach based on disease biology. EC subtypes with high tumor mutational burden (eg, *POLE* mutant/hypermutated and microsatellite instability (MSI)) are highly immunogenic and exhibit more tumor-specific neoantigens, resulting in increased CD3+ and CD8+ tumor-infiltrating lymphocytes and compensatory upregulation of immune checkpoints.^3^ Pembrolizumab, a monoclonal antibody targeting programmed cell death 1 (PD-1), is approved for recurrent MSI-high (MSI-H) tumors including EC based on results from the single-arm phase II KEYNOTE-158 study (57% objective response rate (ORR) in 49 patients with MSI-H EC).^4^ Other agents, such as nivolumab, have shown similar activity in MSI-H EC^5^; however, MSI-H tumors represent only 13%–30% of recurrent ECs and options are required for the microsatellite stable (MSS) population.^1^

As tumor type and accompanying microenvironment-specific contexts drive the expression of multiple inhibitory receptors, discovery efforts have focused on targeting multiple inhibitory receptors unique to the tumor setting to reverse immune system exhaustion and unresponsiveness. Combined immuno-oncology (IO) and antiangiogenic treatment has emerged as a promising strategy, demonstrating synergy between treatment mechanisms.^6^ Antiangiogenic agents have consistently shown signals of activity as treatment for EC,^7^ and the combination of pembrolizumab and lenvatinib (a multiple receptor tyrosine kinase inhibitor (TKI)) was approved by the USA Food and Drug Administration for patients with advanced EC that is not MSI-H or mismatch repair (MMR) deficient and whose disease has progressed following prior systemic therapy.^8^ To date, no biomarkers for response have been identified. Most notably, data are absent on post-IO progression.

Cabozantinib is a multitargeted TKI with potent activity against hepatocyte growth factor receptor (MET), vascular endothelial growth factor (VEGF) receptor 2, RET, and AXL. Single-agent cabozantinib demonstrated response rates of 12%–14% in EC.^9^ Targeting pathways promoting angiogenesis may enhance antitumor immunity and response rates, particularly in MSS EC.^10^

Our translational randomized phase II trial assessed the efficacy and safety of the immune checkpoint inhibitor nivolumab plus cabozantinib versus nivolumab alone in IO-naïve recurrent EC, and the efficacy of the combination in disease that had progressed after IO. Baseline biopsies and serial blood tests for peripheral blood mononuclear cell (PBMC) samples were collected for immune characterization and identification of potential biomarkers of response.

## Methods

### Study design and participants

This open-label randomized phase II trial (NCT03367741), conducted through the National Cancer Institute Experimental Therapeutics Clinical Trials Network, assessed the activity of cabozantinib combined with nivolumab (Arm A) versus nivolumab alone (Arm B) in women with advanced, recurrent, or metastatic EC. Eligibility criteria included Eastern Cooperative Oncology Group (ECOG) performance status 0–2, a diagnosis of measurable disease according to Response Evaluation Criteria in Solid Tumors (RECIST; version 1.1), regardless of the histologic subtype, and radiologic progression after at least one line of previous platinum-based chemotherapy. There was no restriction on the number of prior treatment lines. Patients had to have normal organ and bone marrow function. Exclusion criteria for all arms included: prior cabozantinib treatment; known brain metastases; concomitant treatment with therapeutic doses of anticoagulant; recent bleeding history or tumor invading the gastrointestinal tract; tumor encasing blood vessels; radiographic evidence of cavitating pulmonary lesions; history of bowel obstruction within the preceding 3 months; history of autoimmune disease; or concomitant treatment with steroids within 7 days before the first dose. In each arm, a baseline biopsy (7–28 days before starting treatment) for correlative analysis was mandatory.

Eligible patients were randomized (2:1) to the combination or monotherapy, stratified according to microsatellite status assessed by genomic analysis, or MMR status defined from archival tissue according to local guidelines (online supplemental figure S1). Randomized treatment allocation was assigned centrally using block randomization and a web-based registration system (Oncology Patient Enrollment Network). Randomized codes were provided by an independent statistician. An exploratory cohort (Arm C) included patients previously treated with IO (anti-PD-1, anti-programmed cell death ligand-1 (PD-L1), or anti-PD-L2 therapy, or patients progressing on Arm B) or carcinosarcoma histology. Patients in Arm C received cabozantinib–nivolumab combination therapy.

10.1136/jitc-2021-004233.supp1 Supplementary data

The study protocol was compliant with Good Clinical Practice guidelines and the Declaration of Helsinki. Ethics approval was obtained in the USA and Canada, and for all participating centers. All patients provided written informed consent.

### Procedures

Combination therapy in Arms A and C comprised oral cabozantinib 40 mg/day continuously (days 1–28) and intravenous nivolumab 240 mg (days 1 and 15) in 28-day cycles. Arm B patients received single-agent intravenous nivolumab 240 mg (days 1 and 15) every 28 days. In all arms, the nivolumab dose was increased to 480 mg every 28 days after the first four cycles if deemed tolerable by the treating physician. Response was assessed by CT scan every 8 weeks (±7 days) according to RECIST (version 1.1). Adverse events (AEs) were graded using Common Terminology Criteria for Adverse Events (version 5.0). If patients experienced toxicity, the cabozantinib dose could be reduced one level to 20 mg daily. Nivolumab dose reduction was not permitted.

Patients in Arm B (nivolumab alone) could cross over to cabozantinib–nivolumab combination therapy in Arm C at the time of progression, provided they still met the eligibility criteria for the exploratory post-IO cohort. A biopsy at progression was mandated to analyze changes in the molecular and immunologic landscape after IO treatment. These patients were analyzed in Arm C from the time of crossover.

For translational research, each patient provided one to two fresh needle-core biopsies before starting treatment and whole blood samples at baseline, cycle 1 day 15 (C1D15), and progression. The methodology is described in the online supplemental appendix.

### Outcomes

The primary endpoint was progression-free survival (PFS), defined as the interval between randomization and disease progression or death from any cause, whichever occurred earlier. Secondary endpoints were RECIST-defined ORR, overall survival (OS), and safety. Correlative studies assessed the immune microenvironment in baseline fresh tissue biopsy with mass cytometry (cytometry by time of flight (CyTOF) using a 36-marker immune profiling panel) and potential correlations with clinical outcome.

### Statistical analysis

The trial was designed and powered to detect differences in PFS between treatment Arms A and B. A one-sided log-rank test with an overall sample size of 54 patients (36 in Arm A and 18 in Arm B) would provide 80% power at a 0.10 significance level to detect a HR of 0.50, assuming median OS of 3 months with nivolumab alone. A 5% loss to follow-up was assumed for sample size estimation. The study was anticipated to last for 24 months.

In Arm C, assuming enrollment of 10 patients in the carcinosarcoma cohort and 20 in the post-IO cohort (including patients crossing over from Arm B), the combination regimen was considered to show an activity signal if at least one patient in each cohort achieved a partial response (PR) and one had stable disease (SD) lasting ≥16 weeks.

## Results

### Patient characteristics

**Figure 1:**
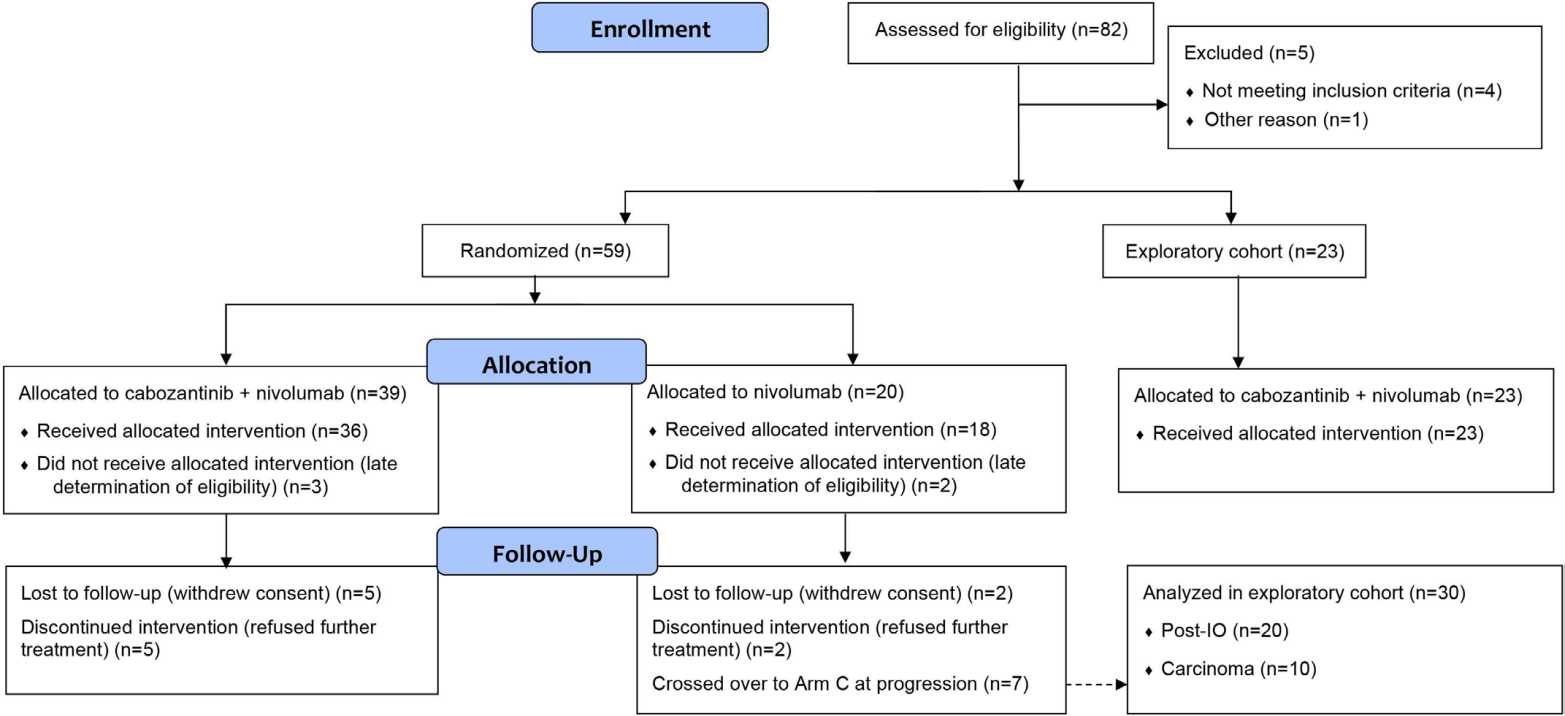
Patient flow. IO, immuno-oncology.

Between January 2018 and December 2019, 82 patients were enrolled from 17 Canadian and US centers. Of these, 77 were treated and evaluable for analysis: 36 in Arm A and 18 in Arm B (figure 1). At the data cut-off (May 5, 2020) at planned study closure, median follow-up was 15.9 months. Table 1 shows key patient characteristics; most patients were heavily pretreated.

**Table 1:** Patient characteristics

Characteristic	Arm A: cabozantinib+ nivolumab (n=36)	Arm B: nivolumab (n=18)	Arm C: cabozantinib+ nivolumab (n=23)
Median age, years (range)	67 (44–77)	66 (41–83)	6 (53–83)
ECOG performance status, n (%)			
0	13 (36)	10 (56)	14 (61)
1	19 (53)	8 (44)	9 (39)
2	4 (11)	0	0
Histology type, n (%)			
Endometrioid	15 (42)	5 (28)	3 post IO (13)
Serous	11 (31) + 1 unevaluable	10 (56) + 2 unevaluable	5 post IO (22)
Clear cell features	5 (14) + 2 unevaluable	0	0
Mixed serous/clear cell/endometrioid	2 (6)	2 (11)	1 post IO (4)
Adenocarcinoma	3 (8)	1 (6)	3 post IO (13)
Carcinosarcoma	0	0	10 + 1 post IO (48)
MMR status, n (%)			
MMR intact	34 (94)	18 (100)	18 (78)
MSI-high	2 (6)	0	5 post IO (22)
Prior treatment lines, n (%)			
2	19 (53)	6 (33)	9 (39)
≥3	17 (47)	12 (67)	14 (61)

ECOG, Eastern Cooperative Oncology Group; IO, immuno-oncology; MMR, mismatch repair system; MSI, microsatellite instability.

### Efficacy

**Figure 2:**
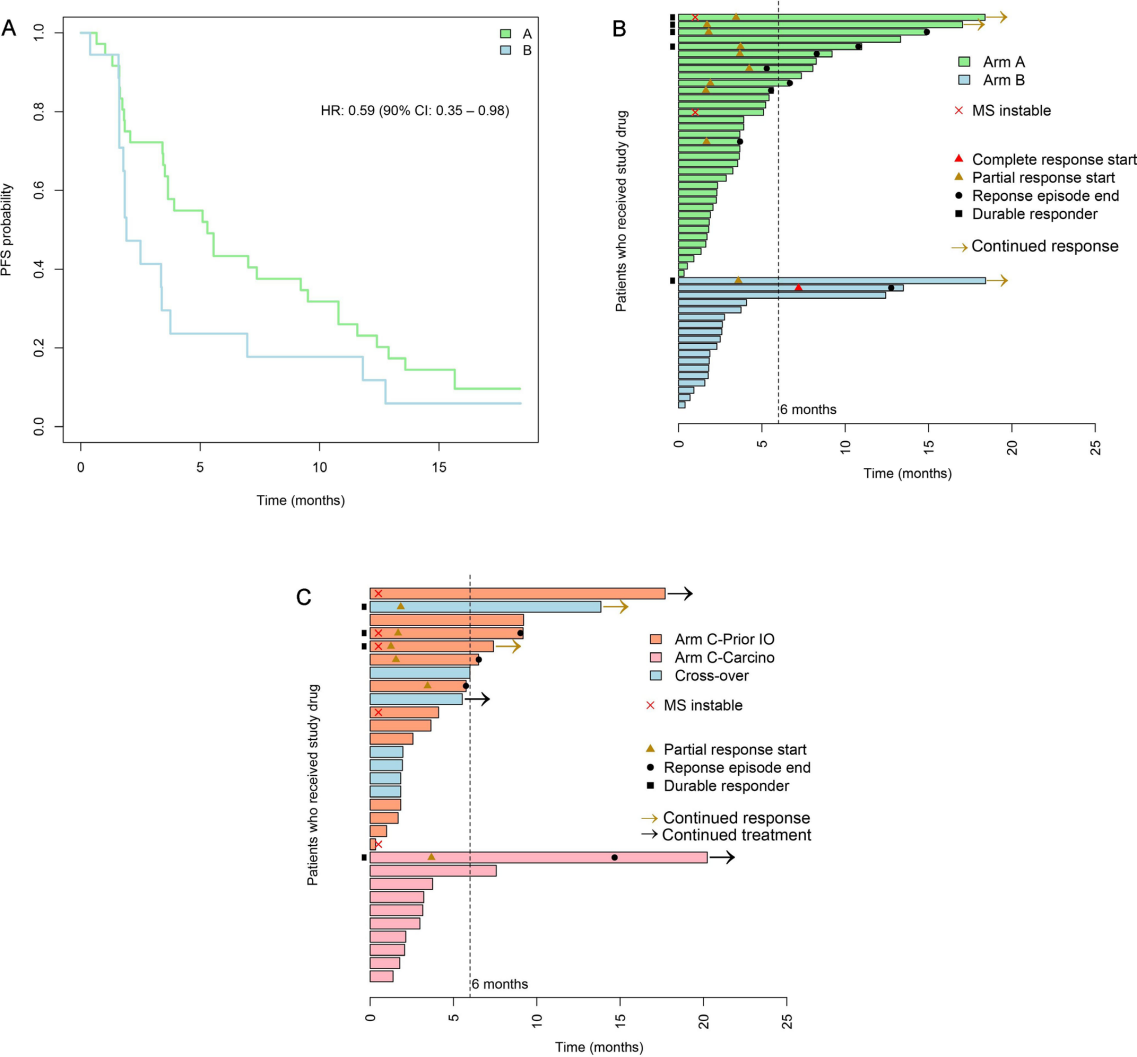
Clinical outcomes. (A) Progression-free survival in Arms A and B. (B) Best response and treatment duration by patient in Arms A and B. (C) Best response and treatment duration in Arm C (carcinosarcoma cohort and post-IO cohort). Carcino, carcinosarcoma; IO, immuno-oncology; MS, microsatellite; PFS, progression-free survival.

PFS was improved with combination therapy (Arm A; median 5.3 months, 90% CI 3.5 to 9.2) versus nivolumab alone (Arm B; median 1.9 months, 90% CI 1.6 to 3.4) (log-rank test p=0.09) (figure 2A). The RECIST ORR was 25% in Arm A versus 11% in Arm B. Best response was disease stabilization in 44% of patients in Arm A versus 11% in Arm B, resulting in overall clinical benefit rates (PR or SD) of 69% versus 22%, respectively (p<0.001) (figure 2B and online supplemental figure S4). Immature OS results (events in 55% of patients), which may be affected by crossover from Arm B to Arm C, showed median OS of 13.0 months (90% CI 10.2 to 18.4) in Arm A and 7.9 months (90% CI 6.1 to not estimable) in Arm B.

In the post-IO treatment cohort (Arm C; n=20), IO rechallenge with cabozantinib–nivolumab resulted in objective responses in five patients (25%) and SD in seven patients (figure 2C). The median duration of SD was 5.5 (range 2.6–17.7) months. In the carcinosarcoma subgroup of Arm C (n=10), one patient had a durable PR (ORR 10%) and five patients had SD, with a median SD duration of 3.2 (range 2.8–7.6) months (figure 2C).

### Safety

Treatment-related toxicities were more frequent with combination therapy than with single-agent nivolumab. The most common treatment-related AEs in Arm A were diarrhea, liver enzyme elevations, fatigue, and hypertension, typically occurring at grade 1/2 intensity except for hypertension (table 2). Serious treatment-related AEs occurred in 11 patients (31%) in Arm A, none of those in Arm B, and 10 patients (33%) in Arm C. Two rare treatment-related and serious AEs occurred with combination treatment: one patient in Arm A experienced grade 4 colonic perforation with grade 5 sepsis and one patient in Arm C experienced grade 5 tumor lysis syndrome. The cabozantinib dose was reduced because of AEs in 44% of patients in Arm A and 52% in Arm C. Treatment was discontinued for AEs in 14% of patients in Arm A (considered probably or possibly related to study treatment each in two patients, and unlikely related to study treatment in one patient), none of those in Arm B, and 17% in Arm C (considered definitely related to study treatment in one patient, probably related in one patient, and possibly related in three patients).

**Table 2:** Most common treatment-related adverse events (any grade in ≥25% of patients or grade ≥3 in ≥10% of patients in any arm; treatment related according to investigator assessment)

Adverse event, n (%)	Arm A (n=36)			Arm B (n=18)			Arm C (n=30)		
	Any grade	Grade 1/2	Grade ≥3	Any grade	Grade 1/2	Grade ≥3	Any grade	Grade 1/2	Grade ≥3
Any	32 (89)	10 (28)	22 (61)	12 (67)	11 (61)	1 (6)	29 (97)	10 (34)	19 (63)
Diarrhea	18 (50)	15 (42)	3 (8)	1 (6)	1 (6)	0	9 (30)	7 (23)	2 (7)
Aspartate aminotransferase increased	17 (47)	15 (42)	2 (6)	0	0	0	16 (53)	14 (47)	2 (7)
Alanine aminotransferase increased	15 (42)	12 (33)	3 (8)	0	0	0	14 (47)	12 (40)	2 (7)
Fatigue	14 (39)	13 (36)	1 (3)	6 (33)	6 (33)	0	16 (53)	14 (47)	2 (7)
Hypertension	11 (31)	5 (14)	6 (17)	0	0	0	10 (33)	7 (23)	3 (10)
Anorexia	11 (31)	11 (31)	0	1 (6)	1 (6)	0	6 (20)	6 (20)	0
Nausea	11 (31)	10 (28)	1 (3)	2 (11)	2 (11)	0	11 (37)	10 (33)	1 (3)
Weight loss	11 (31)	11 (31)	0	1 (6)	1 (6)	0	6 (20)	6 (20)	0
Mucositis oral	11 (31)	9 (25)	2 (6)	1 (6)	1 (6)	0	3 (10)	3 (10)	0
Platelet count decreased	10 (28)	9 (25)	1 (3)	0	0	0	6 (20)	6 (20)	0
Hypothyroidism	8 (22)	8 (22)	0	1 (6)	1 (6)	0	9 (30)	9 (30)	0
Palmar-plantar erythrodysesthesia syndrome	8 (22)	6 (17)	2 (6)	0	0	0	8 (27)	8 (27)	0
Anemia	6 (17)	4 (11)	2 (6)	1 (6)	1 (6)	0	13 (43)	11 (37)	2 (7)
Thyroid-stimulating hormone increased	5 (14)	5 (14)	0	1 (6)	1 (6)	0	8 (27)	8 (27)	0
Lymphocyte count decreased	4 (11)	3 (8)	1 (3)	1 (6)	0	1 (6)	7 (23)	1 (3)	6 (20)

### Immune biomarker analysis

**Figure 3:**
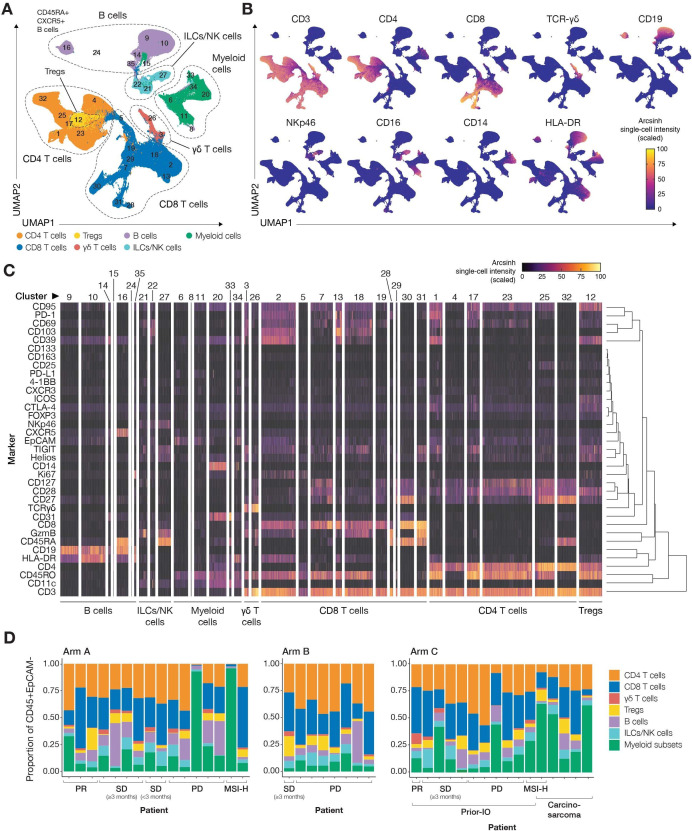
Overview of immune cell populations present in baseline biopsies. CD45+EpCAM– cells from all 40 patients (16 from Arm A (two with MSI-H tumors), 8 from Arm B, and 16 from Arm C (two with MSI-H tumors and five with carcinosarcoma) were pooled for unsupervised clustering using *phenograph*. (A) UMAP visualization shows the distribution of 35 *phenograph*-defined clusters across the major immune cell populations: CD4+ and CD8+ T cells, ɣδ T cells, regulatory T cells (Tregs), B cells, ILCs/NK cells, and myeloid subsets, as shown by the expression of lineage markers. The 35 *phenograph*-defined clusters can be separated into 7 CD4+ T-cell clusters (including one Treg cluster), 10 CD8+ T-cell clusters, two ɣδ T-cell clusters, seven B-cell clusters, three ILC/NK cell clusters, and six myeloid clusters. Each cluster has a unique pattern of marker expression, including markers of immune activation and suppression, cellular adhesion, trafficking, and proliferation. (B) UMAPs show the mean signal intensity and high-dimensional localization of the markers used to define the major immune cell populations. (C) Single-cell heatmap shows the (hierarchical) marker expression profiles that define each of the 35 unique immune cell clusters, grouped by immune cell subsets. (D) The baseline immune composition as a proportion of CD45+ EpCAM– cells is shown for each patient across all treatment Arms. Patients are ordered by best response, MSI status, histology, and study identifier. All major immune populations are present in varying proportions in the baseline biopsies of most patients. CTLA-4, cytotoxic T-lymphocyte antigen-4; EpCAM, epithelial cell-adhesion molecule; HLA, human leukocyte antigen; ILC, innate lymphoid cell; IO, immuno-oncology; MSI, microsatellite instable; MSI-H, microsatellite instable-high; NK, natural killer; PD, progressive disease; PD-1, programmed cell death 1; PD-L1, programmed cell death ligand-1; PR, partial response; SD, stable disease; TCR, T-cell receptor; UMAP, uniform manifold approximation and projection.

Overall, 40 evaluable baseline biopsies were analyzed using a 36-marker CyTOF panel. Unsupervised *phenograph* clustering of pooled CD45++ epithelial cell-adhesion molecule– cells resulted in 35 unique clusters constituting the major immune-cell populations, which were present in baseline biopsies of most patients (figure 3).

**Figure 4:**
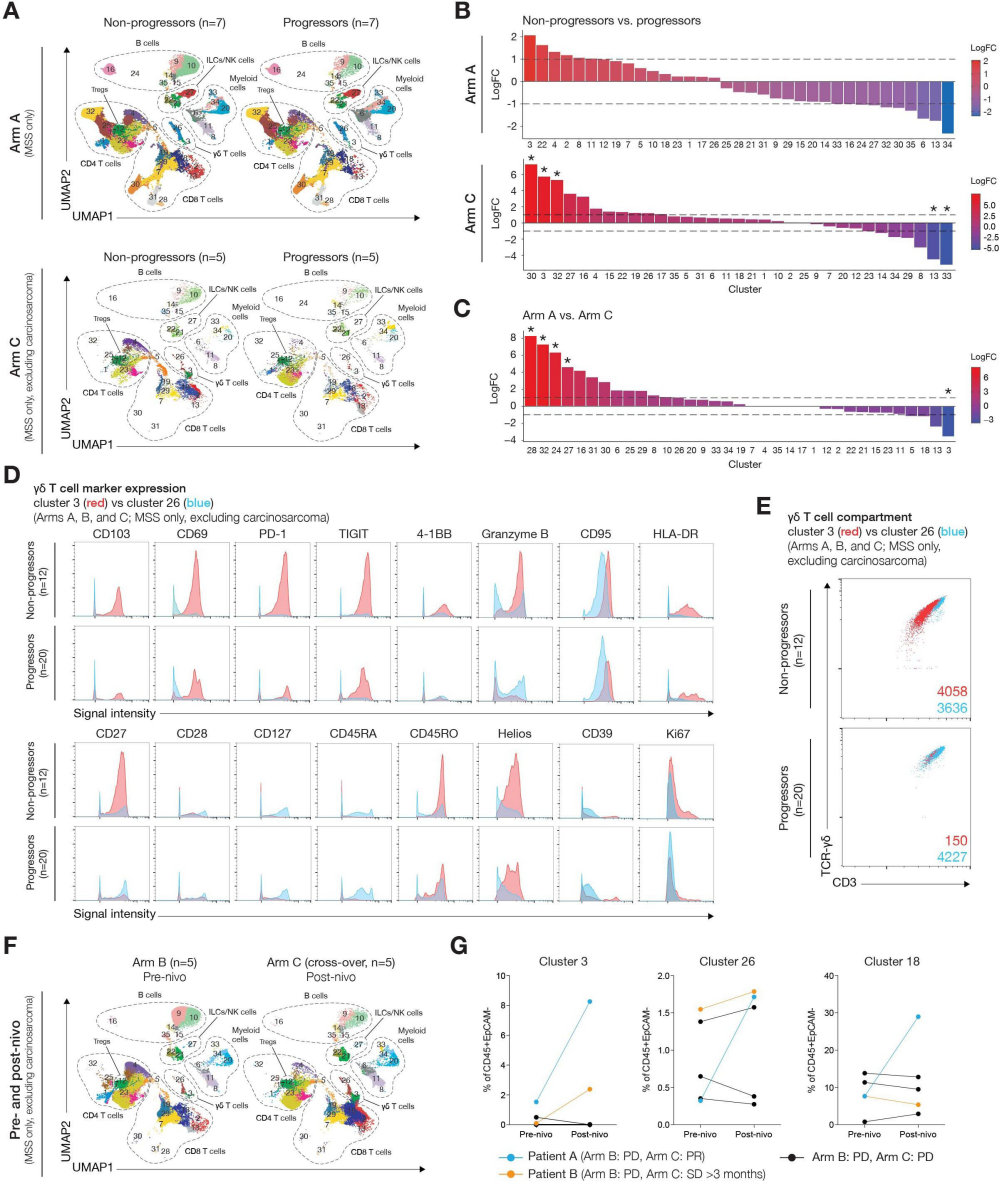
Comparison of baseline biopsies from non-progressors (best response, PR/SD ≥3 months) and progressors (best response, PD/SD <3 months) within Arm A and Arm C. Non-progressors and progressors in Arms A and C included patients with endometrioid, clear cell, and serous histotypes (online supplemental table S2). Patients with MSI-H disease or carcinosarcoma were excluded. (A) UMAP visualizations show the 35 *phenograph*-defined immune cell clusters present in the baseline biopsies of non-progressors and progressors in Arm A (n=7 per group) and Arm C (n=5 per group). (B) Bar graphs show the differential abundance of each immune cell cluster between non-progressors and progressors within Arm A and Arm C. Data are presented as the log fold-change (logFC). LogFC >0 indicates more abundant in non-progressors; logFC <0 indicates less abundant in non-progressors. In Arm A (upper panel), there was no statistically significant difference between non-progressors and progressors, although non-progressors showed a non-significant trend toward a higher proportion (>2 logFC) of cluster 3 ɣδ T cells (3865 cells). In Arm C (lower panel), prior-IO non-progressors had a significantly higher proportion (6 logFC; adjusted p*=*0.009) of the same cluster 3 ɣδ T cells vs progressors. Cluster 30 CD45RA+CD27+ CD8 T cells and cluster 32 CD45RA+CD27+ CD4 T cells were also significantly higher in non-progressors than in progressors; however, we detected only 18 and 30 cells in each cluster, respectively. There were no significant differences between progressors and non-progressors in the proportion of cluster 26 ɣδ T cells. Tissue-resident (CD103+CD69+) GzmB^low^ CD8 T cells (cluster 13; 3894 cells) and CD11c+CD31+ myeloid cells (cluster 33; 149 cells) were significantly more abundant in progressors than non-progressors. (C) Bar graph showing the differential abundance of each immune-cell cluster between Arm A (n=14) and Arm C (n=10). Data are presented as logFC. LogFC >0 indicates more abundant in Arm A; logFC <0 indicates less abundant in Arm A. Several CD45RA+ clusters (clusters 28, 32, 24, and 27) were significantly more abundant in IO-naïve (Arm A) than IO-pretreated (Arm C) patients, whereas activated, tissue-resident (CD103+CD69+) cluster 3 ɣδ T cells were significantly more abundant in patients whose disease progressed on prior IO (see online supplemental figure S5A for CD45RA and CD45RO expression in these clusters; see online supplemental figure S5B for selected marker expression in tissue-resident CD8 T-cell clusters). (D) Histograms show the signal intensity of selected markers on cells from cluster 3 (red) and cluster 26 (blue). Most cluster 3 ɣδ T cells were tissue resident (CD103+, CD69^high^) and PD-1^high^, TIGIT^high^, CD39^high^, and Helios^high^, whereas cluster 26 ɣδ T cells had lower and more variable expression of these T-cell activation/checkpoint markers. (E) Scatter dot plots showing the expression of CD3 and TCR-ɣδ by cluster 3 (red) and cluster 26 (blue) cells in non-progressors (n=12) and progressors (n=20) from Arms A, B, and C. The number of events in each cluster is indicated. Overall, cluster 3 ɣδ T cells were more abundant in baseline biopsies from non-progressors than progressors (4058 vs 150 events), whereas the total number of cluster 26 ɣδ T cells was similar in the two groups (3636 vs 4227 events), suggesting that cluster 3 ɣδ T cells may be important for response to cabozantinib–nivolumab combination therapy. (F) UMAPs show the 35 *phenograph*-defined immune cell clusters present in the baseline biopsies of Arm B crossover patients before treatment with nivolumab (pre-nivo), and at the time of crossover (post-nivo) to Arm C before the start of combination treatment with cabozantinib–nivolumab (n=5). Differential abundance analysis of the *phenograph*-defined clusters revealed no significant differences between pre-nivolumab and post-nivolumab biopsies (online supplemental figure S6A). (G) Graphs depicting the proportion of cells from clusters 18, 3, and 26 pre-nivo and post-nivo among CD45+EpCAM– cells. These paired biopsy data further suggest that an increase in activated tissue-resident cluster 3 ɣδ T cells before cabozantinib–nivolumab combination therapy is potentially associated with a more favorable response. Patient A whose disease progressed on Arm B but responded on Arm C (highlighted in blue) exhibited a 3.8-fold increase in the percentage of activated, tissue-resident (CD103+CD69+) GzmB^high^ CD8+ T cells (cluster 18), and >5-fold increases in the percentage of ɣδ T cells (clusters 3 and 26) following nivolumab monotherapy (see online supplemental figure S6B for all clusters). Compared with the crossover patients whose disease progressed in Arm C, the proportion of clusters 3 and 18 were more than 2-fold higher in patient A post-nivolumab before initiation of combination therapy. Patient B whose disease progressed on Arm B but was stable for ≥3 months on Arm C (highlighted in yellow) had a 25-fold increase in cluster 3 ɣδ T cells following nivolumab monotherapy. *Adjusted p<0.05 by Benjamini-Hochberg method. EpCAM, epithelial cell-adhesion molecule; ILC, innate lymphoid cell; IO, immuno-oncology; nivo, nivolumab; MSS, microsatellite stable; NK, natural killer; PD, progressive disease; PD-1, programmed cell death 1; PR, partial response; SD, stable disease; TCR, T-cell receptor; Treg, regulatory T cell; UMAP, uniform manifold approximation and projection.

In Arm A, differential abundance analysis of the *phenograph*-defined clusters showed no statistically significant differences between non-progressors (best response of PR or SD for ≥3 months) and progressors (best response of progressive disease or SD for <3 months) (figure 4A), although non-progressors showed a trend toward a higher proportion of cluster 3 ɣδ T cells (figure 4B). In Arm C, compared with progressors, prior-IO non-progressors had a significantly higher proportion of the same cluster 3 ɣδ T cells, cluster 30 CD45RA+CD27+ CD8 T cells, and cluster 32 CD45RA+CD27+ CD4 T cells (figure 4B). Tissue-resident (CD103+CD69+) GzmB^low^ CD8 T cells (cluster 13; 3894 cells) and CD11c+CD31+ myeloid cells (cluster 33; 149 cells) were significantly more abundant in progressors than non-progressors. Several CD45RA+ clusters (clusters 28, 32, 24, and 27) were significantly more abundant in IO-naïve than IO-pretreated patients, whereas activated, tissue-resident (CD103+CD69+) cluster 3 ɣδ T cells were significantly more abundant in patients whose disease progressed on prior IO (figure 4C). Most cluster 3 ɣδ T cells were tissue-resident (CD103+, CD69^high^) and PD-1^high^, T-cell immunoglobulin and ITIM domain (TIGIT)^high^, CD39^high^, and Helios^high^, whereas cluster 26 ɣδ T cells had lower and more variable expression of these T-cell activation/checkpoint markers (figure 4D). Overall, cluster 3 ɣδ T cells were more abundant in baseline biopsies from non-progressors than progressors, whereas the total number of cluster 26 ɣδ T cells was similar in the two groups (figure 4E) suggesting that cluster 3 ɣδ T cells may be important for response to cabozantinib–nivolumab combination therapy.

Comparison of baseline biopsies from five Arm B patients with progression on nivolumab monotherapy who subsequently crossed over to Arm C and had a repeat baseline biopsy revealed no significant differences between pre-nivolumab and post-nivolumab biopsies (figure 4F). Paired biopsy data suggest that an increase in activated tissue-resident cluster 3 ɣδ T cells before cabozantinib–nivolumab combination therapy is potentially associated with a more favorable response (figure 4G).

### Comparison of γδ T cells from blood and the tumor microenvironment

**Figure 5:**
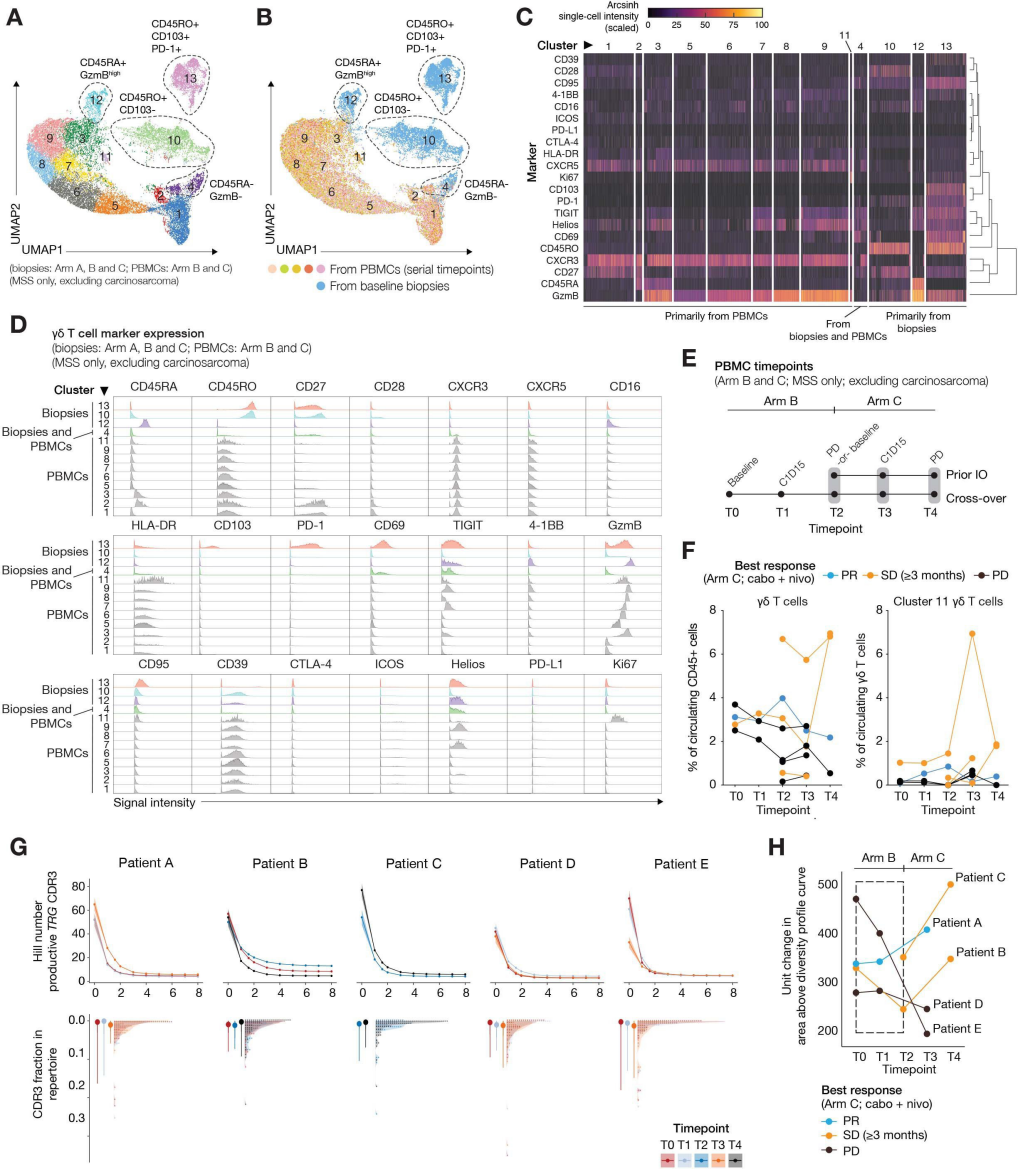
Comparison of ɣδ T cells from baseline biopsies and serial PBMC samples. (A) UMAP shows the 13 *phenograph*-defined ɣδ T-cell clusters present in the baseline biopsies (n=32, Arms A, B, and C) and serial PBMCs (n=28 samples, Arms B and C). (B) UMAP shows the contribution of cells from baseline biopsies (blue) and PBMCs (multiple colors) to each of the 13 *phenograph*-defined ɣδ T-cell clusters. (C) Single-cell heatmap shows the (hierarchical) marker expression profiles that define each of the 13 ɣδ T-cell clusters, grouped by whether the majority of cells in each cluster are from PBMCs, a mix of PBMCs and biopsies, or biopsies. (D) Signal intensity of select markers on ɣδ T cells from each cluster. (E) Schematic shows the time points that serial PBMCs were collected from Arm B and Arm C patients (n=8 patients) for CyTOF analysis. PBMCs were isolated at baseline, C1D15, and at progression. (F) Graphs show the proportions of total ɣδ T cells (left) and cluster 11 ɣδ T cells (right) at each time point. Patients with a best response while on Arm C of PR (blue) and SD ≥3 months (yellow) are highlighted. (G) The top panel shows the diversity profiles (n=14) reconstructed for each patient (n*=*5 patients) at different time points (Baseline T0, Arm B T1, Arm B T2, Arm C T3, time of progression T4). The first three points on each profile demonstrate diversity indices: richness, Shannon diversity, and Gini-Simpson diversity, respectively. The rest of the indices depict the effective number of species when higher weights are given to the most abundant clonotypes. While more linear profiles illustrate more even distribution of clonotypes, high drops in the profile demonstrate skewness of the frequency distribution of clonotypes in the repertoire. Bottom panels add more resolution to the diversity profiles by showing the frequency distribution of each repertoire. Each point demonstrates a unique clonotype with its frequency shown on the y-axis. Repertoires with dots spanning over a wider spectrum of frequencies tend to have larger drops in their diversity profiles. (H) Area above the curve (AAC) of diversity profiles. In the progression window there is either no shift or a decrease in AAC in time points associated with Arm B compared with baseline T0. Comparing AACs of each point inside the progression window with a point in Arm C for each patient shows an increased value of AAC for responders versus a decreased value of AAC for progressors. Patients with microsatellite instability-high disease and those with carcinosarcoma were excluded. C1D15, cycle 1 day 15; Cabo, cabozantinib; CDR3, complementarity-determining region 3; CTLA-4, cytotoxic T-lymphocyte antigen-4; CXCR, C-X-C chemokine receptor; CyTOF, cytometry by time of flight; HLA-DR, human leukocyte antigen – DR isotype; IO, immuno-oncology; MSS, microsatellite stable; nivo, nivolumab; PBMC, peripheral blood mononuclear cell; PD, progressive disease; PD-1, programmed cell death 1; PD-L1, programmed cell death ligand-1; PR, partial response; SD, stable disease; UMAP, uniform manifold approximation and projection.

Profiling of ɣδ T cells from serial PBMC samples from four crossover patients (from Arm B to Arm C) and four whose disease progressed on prior IO (Arm C) resulted in 13 unique clusters (figure 5A). Cluster 10, 12, and 13 ɣδ T cells were primarily from baseline biopsies, whereas cluster 4 ɣδ T cells were from both biopsies and PBMCs (figure 5B). The remaining clusters comprised primarily peripheral ɣδ T cells, suggesting that circulating and tumor biopsy ɣδ T cells have different marker expression profiles and phenotypes. CD45RO^high^, CD103+CD69+PD-1+, and CD95^high^ ɣδ T cells were detected only in biopsies (cluster 13), while CD39, granzyme B, TIGIT, and Helios were expressed in clusters of ɣδ T cells from biopsies and PBMCs (figure 5C, D). C-X-C chemokine receptor (CXCR)3 and CXCR5 were expressed primarily in peripheral ɣδ T cells, and cluster 11 ɣδ T cells were Ki67+.

There were no significant changes in the proportion of peripheral ɣδ T cells at C1D15 (T1) of nivolumab monotherapy, or at C1D15 of cabozantinib–nivolumab combination therapy (T3; figure 5E, F). The proportion of Ki67+ ɣδ T cells (cluster 11) increased in patient A during progression on Arm B (T2), then decreased at T3. In contrast, Ki67+ ɣδ T cells (cluster 11) increased at T3 in six of the seven remaining patients.

### γδ T-cell repertoire diversity analysis

When comparing the diversity profiles of the systemic ɣδ repertoire in treatment-naïve patients at T0 versus Arm B T1 time points (patients A, D, and E), the two curves are either superimposed (illustrating a lack of hierarchical clonotype distribution shifts in systemic ɣδ repertoire) or show a slight upward shift from T0 to T1 (figure 5G). A more pronounced upward shift is seen from T0 to Arm B T2 (patient B). Within the progression window (from T0 to T2, during which all patients in Arm B had progressive disease), there were either no significant shifts in the area above the diversity profile curve (AAC) or a decrease in AAC through the progression zone (figure 5H). As the patients entered Arm C and started combination treatment, the AAC shifts distinguished responders from non-responders. Patients whose disease continued to progress in Arm C showed a sustained descending AAC trend over time. These patients also had a more linear diversity profile in T3, demonstrating increased evenness in their clonotypic frequency distribution, whereas patients who started to respond in Arm C demonstrated an ascending AAC trend out of the progression window. Characterization of the T-cell receptor (TCR) γ VJ gene segment is shown in online supplemental figure S7.

## Discussion

To the best of our knowledge, this is the first study demonstrating benefit from cabozantinib–nivolumab combination therapy in patients with heavily pretreated recurrent EC. These results confirm the benefit of combining antiangiogenic agents and IO, as observed with pembrolizumab and lenvatinib in a less pretreated population.^9^

Our results were broadly consistent with those recently reported from the phase III KEYNOTE-775 trial of pembrolizumab–lenvatinib combination therapy (median PFS of 6.6 months, median OS of 17.4 months, RECIST ORR of 32%)^11^ given the differences in the patient populations. Our study enrolled more heavily pretreated patients (predominantly ≥3 prior regimens) and included patients with ECOG performance status 2 and only two patients with MSI-H EC. ORR increased with the cabozantinib–nivolumab combination versus nivolumab alone and appeared higher than in previous reports of single-agent cabozantinib in a similar patient population (12%–14%),^9^ yet we cannot directly assess the additive value of cabozantinib in this subgroup. In KEYNOTE-775, grade ≥3 treatment-related AEs occurred in 89% of patients receiving pembrolizumab–lenvatinib combination therapy compared with 64% with the cabozantinib–nivolumab combination in our study. Further investigation is required to define patient-reported outcomes and potential preventive measures to help with the management of AEs.

A limitation of our study may be the control arm choice of nivolumab alone rather than standard chemotherapy. However, the expected response to single-agent doxorubicin or weekly paclitaxel is ~10%–15%,^1 11^ similar to that observed with nivolumab alone. Our trial was designed as a translational study exploring the potential to improve response to immunotherapy, particularly in a heavily pretreated population with no standard-of-care options available. In the recent non-randomized phase I GARNET study, the ORR was 13% with single-agent dostarlimab in the MSS cohort.^12^ To date, no information on molecular profiling is available from the GARNET study. Another potential criticism is the observed imbalance in prior therapy: patients randomized to Arm B (nivolumab alone) were more heavily pretreated than those randomized to Arm A (combination therapy). This may lead to bias when assessing the contribution of cabozantinib to clinical outcomes. In addition, there is a risk that patients may have crossed over to combination therapy on the basis of pseudoprogression rather than true progression on single-agent nivolumab. This could complicate analysis of patients who crossed over and subsequently responded to combination therapy. Pseudoprogression has been described in the literature in nivolumab-treated patients with ovarian cancer.^13 14^ However, evidence is limited and some reports suggest that pseudoprogression with nivolumab may be less common than initially suspected, at least in some tumor types.^15 16^ Long-term OS results from the present study may provide further insight.

A strength of our study is the inclusion of specific understudied populations of patients with progression after IO or with aggressive poor-prognosis carcinosarcoma, who have typically been excluded from clinical trials. Cabozantinib–nivolumab combination therapy showed an encouraging preliminary signal of activity in carcinosarcoma histology, for which current treatment options are very limited, and our data also suggest that resistance to IO may be overcome by combining cabozantinib with nivolumab. To our knowledge, this is the first pilot study to assess treatment in the post-IO setting, to investigate mechanisms of therapeutic resistance, and to offer a potential treatment option following progression on IO.

CyTOF analysis of fresh baseline biopsies provides the first high-dimensional insight into the immune microenvironment of recurrent EC. Unsupervised *phenograph* clustering resulted in 35 unique immune-cell subsets including subsets of CD8+ T cells (clusters 2, 13, and 18), which differ in their expression of CD103, CD69, and PD-1, as well as Ki67 and human leukocyte antigen—DR isotype (both higher in cluster 2; see online supplemental figure S5B), CD39 (higher in clusters 2 and 13), granzyme B (higher in cluster 18), and ɣδ T cells (cluster 3), which together may be targets of PD-1 blockade. The frequency of tissue-resident ɣδ T cells has shown an association with favorable outcomes in a pan-cancer meta-analysis of gene expression signatures across 39 malignancies.^17^ We also identified a higher proportion of highly activated, tissue-resident ɣδ T cells (cluster 3) in patients whose disease progressed on IO but benefited from IO rechallenge (nivolumab) combined with cabozantinib. We observed no significant differences in cluster 26 ɣδ T cells, which have lower CD103 and CD69 expression, suggesting that the abundance of cluster 3 ɣδ T cells at baseline may be uniquely associated with clinical benefit from cabozantinib–nivolumab combination therapy in IO-pretreated patients. ɣδ T cells play important roles in both protumor and antitumor immunity. In a chemically induced tumor model, ɣδ T cells were shown to be protective by killing tumor cells in a natural killer group 2 type D-dependent manner^18^ and by producing interferon-ɣ early in the tumor microenvironment.^19^ Conversely, the pro-tumor functions of ɣδ T cells centralize on interleukin (IL)-17 production, which can help recruit neutrophils and polymorphonuclear myeloid-derived suppressor cells to limit αβ T-cell function.^20–22^ Additionally, IL-17 made by ɣδ T cells can promote angiogenesis via the secretion of VEGF and angiopoietin-2 by tumor cells and macrophages.^23 24^ Whether cabozantinib plays a role in dampening ɣδ T cell-mediated angiogenesis should be explored further. Additional studies are required to expand this immune profiling to larger cohorts of patients with advanced EC to evaluate further the prognostic potential of activated, tissue-resident ɣδ T cells in IO settings. Although marker expression profiles differ between peripheral and tissue-resident ɣδ T cells, further TCR analysis in circulating ɣδ T cells may reveal potential associations between TCR diversity and response to IO.

In conclusion, our study confirmed the benefit of combining IO and antiangiogenic agents for the treatment of recurrent EC, demonstrating antitumor activity and tolerability even in heavily pretreated patients and patients with carcinosarcoma. Furthermore, our study showed the potential benefit of this combination in a subset of patients previously exposed to IO, which merits further investigation.

## Data Availability

All data relevant to the study are included in the article or uploaded as supplementary information.
