# Supplementary material for: Translational randomized phase II trial of cabozantinib in combination with nivolumab in advanced, recurrent, or metastatic endometrial cancer
Source: J Immunother Cancer. 2022 Mar 14;10(3):e004233. doi: 10.1136/jitc-2021-004233 (PMC8921950; doi:10.1136/jitc-2021-004233)
Supplement: Supplementary data [file jitc-2021-004233supp001.pdf]

A translational randomized phase II trial of cabozantinib in combination with nivolumab in advanced, recurrent, or metastatic endometrial cancer

Stephanie Lheureux et al

ONLINE SUPPLEMENTAL APPENDIX

TRANSLATIONAL RESEARCH METHODS

Core biopsies and peripheral blood mononuclear cells

Before the start of treatment, one to two fresh needle-core biopsies were obtained and shipped overnight in Roswell Park Memorial Institute (RPMI)-1640 medium (catalog #11875093; ThermoFisher, Waltham, USA). Biopsies were minced into fragments and enzymatically digested for 30 minutes at 37°C into single-cell suspensions using the gentleMACS Octo dissociator and human tumor dissociation kit (catalog #130-095-929; Miltenyi Biotec, Bergisch Gladbach, Germany) following the manufacturer’s protocols. Whole blood was collected from patients at baseline, cycle 1 day 15, and at progression. Samples were shipped overnight at ambient temperature and peripheral blood mononuclear cells (PBMCs) were isolated by Ficoll gradient centrifugation. Briefly, whole blood was centrifuged at 1500 rpm for 10 minutes at room temperature (no brake; TX-750 rotor, Sorvall ST40R centrifuge, ThermoFisher). The cellular component was resuspended in phosphate-buffered saline (PBS; catalog #10-010-031; ThermoFisher), overlayed onto a Ficoll gradient (catalog #17-5442-03; GE Healthcare, Chicago, USA) and centrifuged at 1000 rpm for 25 minutes at room temperature; increasing to 1800 rpm after 5 minutes (settings; acceleration = 9, deceleration = 3). PBMCs were collected, and any excess red blood cells were removed using ammonium-chloride-potassium lysis buffer (catalog #118-156-721; Quality Biological, Gaithersburg, USA). Single-cell suspensions and PBMCs were viably frozen in RPMI-1640 medium with 10% dimethyl sulfoxide (catalog #BP231-100; ThermoFisher) containing 12.5% human serum albumin (catalog #800-125P; Gemini Bio, West Sacramento, USA), and cryopreserved in liquid nitrogen before analysis.

CyTOF staining and analysis

Viably frozen single-cell suspensions from biopsy samples and PBMCs were thawed and stained with a 36-marker panel (online supplemental table S1). Viably frozen single-cell suspensions from biopsy tissues were stained in 10 batches following the method described by Gadalla et al.[1] PBMCs were stained in four batches. PBMCs isolated from a healthy donor (IRB #11-0343) were stained in parallel with each batch of patient samples and used as a control for batch correction. Similarly, PBMCs isolated from a fresh leukopack (catalog #70500; STEMCELL Technologies, Vancouver, Canada) were used as a control for the clinical PBMC samples. PBMC samples were barcoded using Cell-ID 20-Plex Pd Barcoding kit (catalog #201060; Fluidigm, South San Francisco, USA) in groups of six to seven samples per batch. Cells were filtered and cytometry by time of flight (CyTOF) data were acquired at the SickKids-UHN Flow and Mass Cytometry Facility on a third-generation Helios mass cytometer (Fluidigm).

Standard CyTOF data-cleaning steps were performed manually using CytoBank (catalog #C47384; Beckman Coulter, Brea, USA) to exclude cell aggregates, debris, and dead cells, as previously described.[2] CD45+epithelial cell-adhesion molecule(EpCAM)- cells were gated out for further analysis. The total number of CD45+EpCAM- cells acquired ranged from 152 to 74,644 cells/sample, with an average of 12,919 cells/sample. PBMC samples were de-barcoded manually using FlowJo Software (version 10.6 for Mac; BD Biosciences, Franklin Lakes, USA). CD45+ cells were gated out for further analysis. Average number of CD45+ cells was 36,000 cells/sample.

Using the *CytofBatchAdjust* R package,[3] technical and instrumental variation between batches was normalized to the 90th percentile using PBMCs from a healthy donor as an anchor sample within each batch (online supplemental figure S2).[4] Subsequently, unsupervised computational clustering was performed on the normalized-arcsin transformed data using the *phenograph* algorithm and *Rphenograph* R package (V0.99.1).[5] C-X-C chemokine receptor 3 (CXCR3), cytotoxic T-lymphocyte antigen-4 (CTLA-4), Helios, programmed cell death ligand-1 (PD-L1), CD133, CD31, and CD95 were excluded from clustering because they did not vary in the dataset and had no effect in driving the clustering. Excluding markers with low variation makes clustering less computationally exhaustive. The distinct phenotype of each cluster was visualized in a heatmap generated using the *ggplot2* R package. Uniform manifold approximation and projection (UMAP), using the *umap* R package (V0.2.4.1) was performed to compress and visualize the high-dimensional data and cell clusters in two dimensions.[6] To accommodate the large number of cells collected from PBMC samples relative to biopsies, and to meet the computational demands to analyze such a large single-cell dataset, we employed a faster *phenograph* algorithm (*Fast-PG* R package) for cell clustering,[7] and used R implementation of the “umap-learn” algorithm from the *umap* R package for dimensionality reduction.[6]

The differential abundance of immune cell clusters between patient groups was determined using the diffcyt-DA-edgeR method from the *diffcyt* R package (V1.2.23). The edgeR method normalizes the number of cells per cluster to the total number of cells per sample to enable cluster abundance comparisons between groups.[8] The results of the differential abundance tests were expressed as log fold-change

between groups. P values were adjusted using Benjamini–Hochberg method to control for false discovery rate.

Cell sorting

Cryopreserved PBMCs were thawed and washed with RPMI-1640 medium containing 10% fetal bovine serum (FBS) and 100 µg/mL DNase I (catalog #DN25-100MG; Sigma-Aldrich, St. Louis, USA). Cells were incubated in PBS with Fc Block (catalog #564220; BD Biosciences) and eFluor 506 fixable viability dye (catalog #65-0866-18; ThermoFisher) for 30 minutes at 4°C. Cells were then stained in fluorescence-activated cell-sorting (FACS) buffer (2% FBS in PBS) with anti-CD4-fluorescein isothiocyanate (1:20; clone RPA-T4; BD Biosciences), anti-CD8a-APC (1:100; clone RPA-T8; ThermoFisher), anti-CD3-PE-Cy7 (1:100; clone UCHT1; ThermoFisher), and anti-T-cell receptor(TCR)γδ-PE (1:50; clone B1.1; ThermoFisher) for 30 minutes at 4°C. Finally, stained cells were washed twice with FACS buffer, filtered, and sorted with a FACSAria Fusion Flow Cytometer (BD Biosciences).

TCR hybrid capture sequencing and repertoire diversity analysis

We applied the CapTCR-seq hybrid capture protocol[9] to genomic DNA libraries of flow-sorted ɣδ T cells collected from serial PBMCs from timepoints T0, T1, T2, T3, and T4 (n=26). However, we had to exclude 12 samples due to their low yield. TCR γ gene (*TRG*) complementarity-determining region 3s (CDR3s) were recovered from FASTQ files using MiXCR software (V3.0.12)[10] with default parameters for RNA sequencing data processing (-p rna-seq -s hsa). As the robustness of diversity indices is influenced by sample saturation and could be biased in unsaturated samples, we then calculated the completeness of the repertoire to ensure

comparison of samples of equivalent saturation to prevent inaccurate comparisons between diversity signatures of different samples. To define a mathematical completeness index, the changes in total number of CDR3s as a function of total sequencing reads were modeled (using *mgcv* R package) and the first derivatives of the curves were calculated (using *gratia* R package). Once the confidence intervals of the first derivative curves crossed zero and remained on a plateau, samples were assumed to have full mathematical coverage. All 14 samples represented in the study were mathematically saturated.

The diversity of the TRG repertoire was analyzed using Hill numbers suggested by the framework iNterpolation/EXTrapolation (iNEXT) (using *iNEXT* R package).[11] The area above the diversity profile curve (AAC) for each diversity profile was then calculated using the *MESS* R package. However, calculating the significance of the AAC shifts was not possible due to the small size of the analyzed samples. VJ gene segment pairing was analyzed following the pipeline suggested by Dash et al.[12]

Analysis of immune biomarkers

Viably frozen single-cell suspensions from fresh baseline biopsies were stained with a 36-marker CyTOF panel. CD45+EpCAM- cells from all patients across each treatment arm were pooled for unsupervised clustering using *phenograph*. To identify immune cell populations that might be associated with clinical benefit from cabozantinib and nivolumab combination therapy, we compared the baseline immune profile of non-progressors (best response of partial response or stable disease lasting for ≥3 months) and progressors (best response of progressive disease or stable disease lasting for <3 months) in Arms A and C. Non-progressors and progressors in Arm A and C included patients with endometrioid, clear cell, and

serous histotypes (online supplemental table S2). Patients with microsatellite instability-high disease were excluded from this analysis to allow focus on groups of microsatellite stable cases from each arm. Patients with carcinosarcoma, which has a different biology, were also excluded from the Arm C analysis.

Comparison of ɣδ T cells from blood and the tumor microenvironment

We used CyTOF to profile the ɣδ T cells from serial PBMC samples from patients who crossed over from Arm B to Arm C and patients whose disease had progressed on prior IO (Arm C) to assess potential effects of nivolumab monotherapy and cabozantinib plus nivolumab combination therapy on circulating ɣδ T cells. We pooled the ɣδ T cells (live, CD45+CD3+TCR-ɣδ+) from both PBMC and biopsy samples and subjected the cells to unsupervised clustering using *phenograph*. Circulating ɣδ T cells were also assessed at serial time points (T0–T4).

ɣδ T-cell repertoire diversity analysis

To capture the changes in the repertoire diversity of systemic ɣδ T cells following enrollment in Arm B and Arm C, we performed bulk TCR sequencing[9] on flow-sorted ɣδ T cells from PBMCs. PBMCs were serially collected from four crossover patients (from Arm B to Arm C) and one patient who progressed on prior IO (Arm C). To quantitatively explain TCR diversity and clonality we constructed diversity profiles for each sample,[13] which is a continuum of Hill numbers with varying orders of q (online supplemental figure S3).

REFERENCES

1. Gadalla R, Noamani B, MacLeod BL, *et al.* Validation of CyTOF against flow cytometry for immunological studies and monitoring of human cancer clinical trials. *Front Oncol* 2019;9:415.
2. Lee BH, Rahman AH. Acquisition, processing, and quality control of mass cytometry data. *Methods Mol Biol* 2019;1989:13–31.
3. R Core Team. R: a language and environment for statistical computing. R Foundation for Statistical Computing, Vienna, Austria, 2016. URL http://www.R-project.org/
4. Schuyler RP, Jackson C, Garcia-Perez JE, *et al.* Minimizing batch effects in mass cytometry data. *Front Immunol* 2019;10:2367.
5. Levine JH, Simonds EF, Bendall SC, *et al.* Data-driven phenotypic dissection of AML reveals progenitor-like cells that correlate with prognosis. *Cell* 2015;162:184–197.
6. Becht E, McInnes L, Healy J, *et al.* Dimensionality reduction for visualizing single-cell data using UMAP. *Nat Biotechnol* 2019;37:38–44.
7. Bodenheimer T, Halappanavar M, Jefferys S, *et al.* FastPG: fast clustering of millions of single cells. *bioRxiv* 2020;2006.2019.159749. doi:10.1101/2020.06.19.159749
8. Weber LM, Nowicka M, Soneson C, Robinson MD. diffcyt: differential discovery in high-dimensional cytometry via high-resolution clustering. *Commun Biol* 2019;2:183.
9. Malignant melanoma of the skin. *Drug Ther Bull* 1988;26:73–75.
10. Bolotin DA, Poslavsky S, Mitrophanov I, *et al.* MiXCR: software for comprehensive adaptive immunity profiling. *Nat Methods* 2015;12:380–381.

11. Hsieh T, Ma KH, Chao A. iNEXT: an R package for rarefaction and extrapolation of species diversity (Hill numbers). *Methods Ecol Evolution* 2016;7:1451–1456.

12. Dash P, Fiore-Gartland AJ, Hertz T, *et al.* Quantifiable predictive features define epitope-specific T cell receptor repertoires. *Nature* 2017;547:89–93.

13. Chao A, Chiu C-H, Jost L. Unifying species diversity, phylogenetic diversity, functional diversity, and related similarity and differentiation measures through Hill numbers. *Annu Rev Ecol Evol Syst* 2014;45:297–324.

Online Supplemental Table S1. CyTOF immune profiling panel

Channel	Marker	Clone	Custom conjugation (Y/N)[a]	Vendor	Catalog number
89 Y	CD45	HI30	N	Fluidigm	3089003B
141 Pr	CD45RA	HI100	Y	BioLegend	304239
142 Nd	HLA-DR	L243	Y	BioLegend	307651
143 Nd	ICOS	DX29	Y	BD Biosciences	557801
145 Nd	CXCR3	G025H7	Y	BioLegend	353733
146 Nd	CD8[a]	RPA-T8	Y	BioLegend	301053
147 Sm	CD4	RPA-T4	Y	BioLegend	300541
148 Nd	EpCAM[b]	9C4	Y	BioLegend	324229
149 Sm	FOXP3	236A/E7	Y	ThermoFisher	14-4777-82
150 Nd	CD103	B-Ly7	Y	ThermoFisher	14-1038-82
151 Eu	CD39	A1	Y	BioLegend	328221
152 Sm	CD11c	Bu15	Y	BioLegend	337221
153 Eu	CD3	UCHT1	Y	BioLegend	300443
154 Sm	CD69	FN50	Y	BioLegend	310939
155 Gd	CD45RO	UCHL1	Y	BioLegend	304239
156 Gd	CD14	M5E2	Y	BioLegend	301843
158 Gd	CD27	O323	Y	BioLegend	302839
159 Tb	CD19	HIB19	Y	BioLegend	302247
160 Gd	CD25	M-A251	Y	BioLegend	101913
161 Dy	Ki67	Ki-67	Y	BioLegend	350523
162 Dy	CD28	CD28.2	Y	BioLegend	302937
163 Dy	4-1BB	4B4-1	Y	BioLegend	Custom (carrier-free)
164 Dy	Granzyme B	GB11	Y	BioLegend	Custom (carrier-free)
165 Ho	PD-1	EH12.2H7	Y	BioLegend	329941
166 Er	CD31[b]	WM59	Y	BD Biosciences	550389
166 Er	TIM3[c]	F38-2E2	Y	BioLegend	345019
167 Er	CD95	DX2	Y	BioLegend	305631
168 Er	CXCR5	MU5UBEE	Y	ThermoFisher	14-9185-82
169 Tm	TCR-γδ	5A6.E9	Y	N/A (hybridoma)[d]	N/A
170 Er	CTLA-4	14D3	Y	ThermoFisher	14-1529-82

**Online Supplemental Table S1.** CyTOF immune profiling panel					
**Channel**	**Marker**	**Clone**	**Custom conjugation (Y/N)[a]**	**Vendor**	**Catalog number**
171 Yb	Helios	22F6	Y	BioLegend	Custom (carrier-free)
172 Yb	CD127	RDR5	Y	ThermoFisher	14-1278-82
173 Yb	NKp46	9E2	Y	BD Biosciences	557911
174 Yb	TIGIT	MBSA43	Y	ThermoFisher	16-9500-82
175 Lu	PD-L1	29E.2A3	Y	BioLegend	329719
176 Yb	CD133[b]	Clone 7	Y	BioLegend	372802
176 Yb	LAG3[c]	7H2C65	Y	BioLegend	Custom (carrier-free)
191 Ir	DNA/Cell ID	N/A	N	Fluidigm	201192B
193 Ir	DNA/Cell ID	N/A	N	Fluidigm	201192B
195 Pt	Cisplatin	N/A	N	BioVision	1550
209 Bl	CD16	3G8	N	Fluidigm	3209002B

Vendors: BD Biosciences, NJ, USA; BioVision, CA, USA; BioLegend, CA, USA; Fluidigm, CA, USA; ThermoFisher Scientific, MA, USA.

CTLA-4, cytotoxic T-lymphocyte antigen-4; CXCR, C-X-C chemokine receptor; CyTOF, cytometry by time of flight; EpCAM, epithelial cell-adhesion molecule; HLA-DR, human leukocyte antigen – DR isotype; LAG3, lymphocyte activation gene-3; N/A, not applicable; PD-1, programmed cell death-1; PD-L1, programmed cell death ligand-1; TCR, T-cell receptor; TIGIT, T-cell immunoglobulin and ITIM domain.

[a]Custom conjugations were carried out by the SickKids-UHN Flow and Mass Cytometry Facility using the Multimetal Maxpar kit (Fluidigm) following the manufacturer’s instructions.

[b]148Nd-EpCAM, 166Er-CD31, and 176Yb-CD133 were only used to stain single-cell suspensions from biopsies.

[c]166Er-TIM3 and 176Yb-LAG3 were only used to stain peripheral blood mononuclear cells.

[d]TCR-ɣδ antibody was provided by Dr Cynthia Guidos (Hospital for Sick Children Research Institute and University of Toronto).

Online Supplemental Table S2. Tumor histology of patients included for cytometry by time of flight analysis

Arm	Response group	Histology		
		Endometrioid	Serous	Clear cell
A	Non-progressors (n = 7)	3	3	1
	Progressors (n = 7)	5	1	1
C	Non-progressors (n = 5)	2	3	0
	Progressors (n = 5)	3	2	0

Online Supplemental Figure S1. Study schema. CT, computed tomography; ECOG PS, Eastern Cooperative Oncology Group performance status; IV, intravenously; MSI, microsatellite instable; MSS, microsatellite stable; PFS, progression-free survival; PO, orally; q2w, once every 2 weeks; q4w, once every 4 weeks.

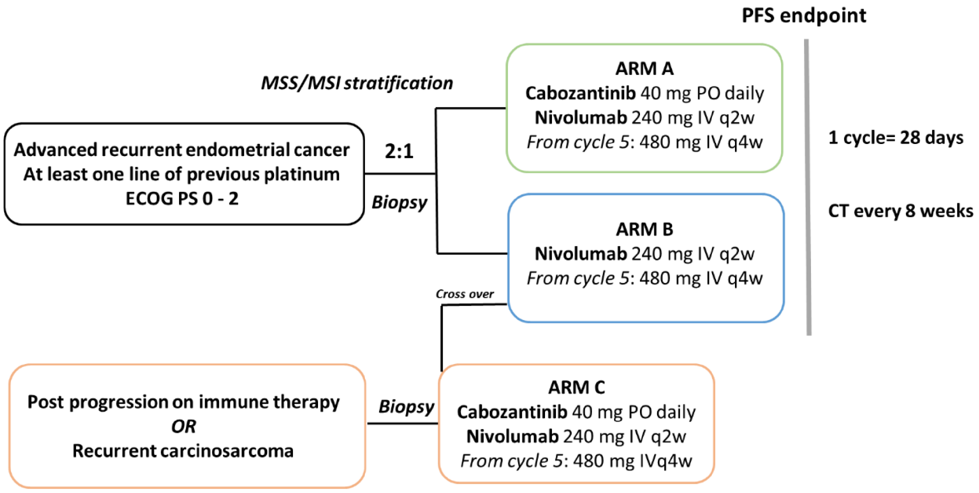

Online Supplemental Figure S2. Variability in mean signal intensity per channel for CyTOF batch normalization to the 90th percentile. Mean signal intensity of each marker for all single-cell events from each healthy donor PBMC (anchor) replicate pre- and post-permutation for biopsy (A) and PBMC (C) panels. Variance of mean signal intensities for these same markers pre- and post-permutation for biopsy (B) and PBMC (D) panels. CTLA-4, cytotoxic T-lymphocyte antigen-4; CXCR, C-X-C chemokine receptor; CyTOF, cytometry by time of flight; HLA-DR, human leukocyte antigen – DR isotype; LAG3, lymphocyte activation gene-3; PBMC, peripheral blood mononuclear cell; PD-1, programmed cell death-1; PD-L1, programmed cell death ligand-1; TIGIT, T-cell immunoglobulin and ITIM domain.

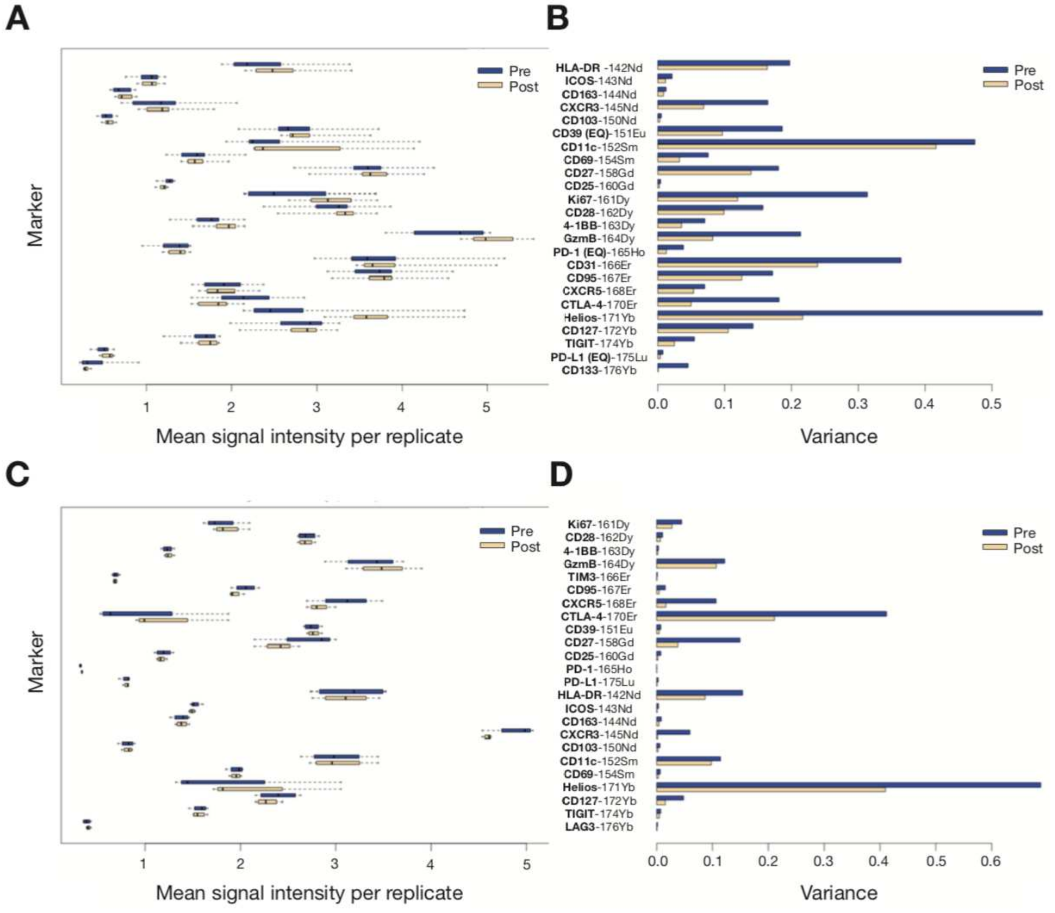

Online Supplemental Figure S3. Interpretation of T-cell receptor diversity graphs. A diversity profile is a continuum of Hill numbers with varying orders of q. The parameter q can be used to emphasize or de-emphasize the weight of abundant or rare clonotypes, ie, it dictates the sensitivity of the index to common and rare species. $q=0$ is completely insensitive to clonal frequency; $0<q<1$ applies more weight on the rare clonotypes; and $q>1$ puts more weight on the high-frequency clonotypes. Thus, when q is low, the Hill numbers are mostly determined by the presence or absence of clonotypes, whereas when q is high, only the most frequent clonotypes matter. For $q\rightarrow 1$ (Shannon diversity), all clonotypes are weighted by their frequencies and neither of the rare or dominant clones are favored. As q increases, all the clonotypes, except for dominant ones, are discounted and the resulting value can be interpreted as the effective number of dominant clonotypes in the sample. For samples with heterogeneous clone frequency, the big drops in the diversity profiles as q increases indicate a high degree of dominance of a few clonotypes in the sample (Repertoire III). If the samples had been completely without dominance (a theoretically even sample with S equally common clonotypes with all species equally likely), all values of q would return the same value of Hill number and thus the repertoire would have a more linear diversity profile (repertoire I).

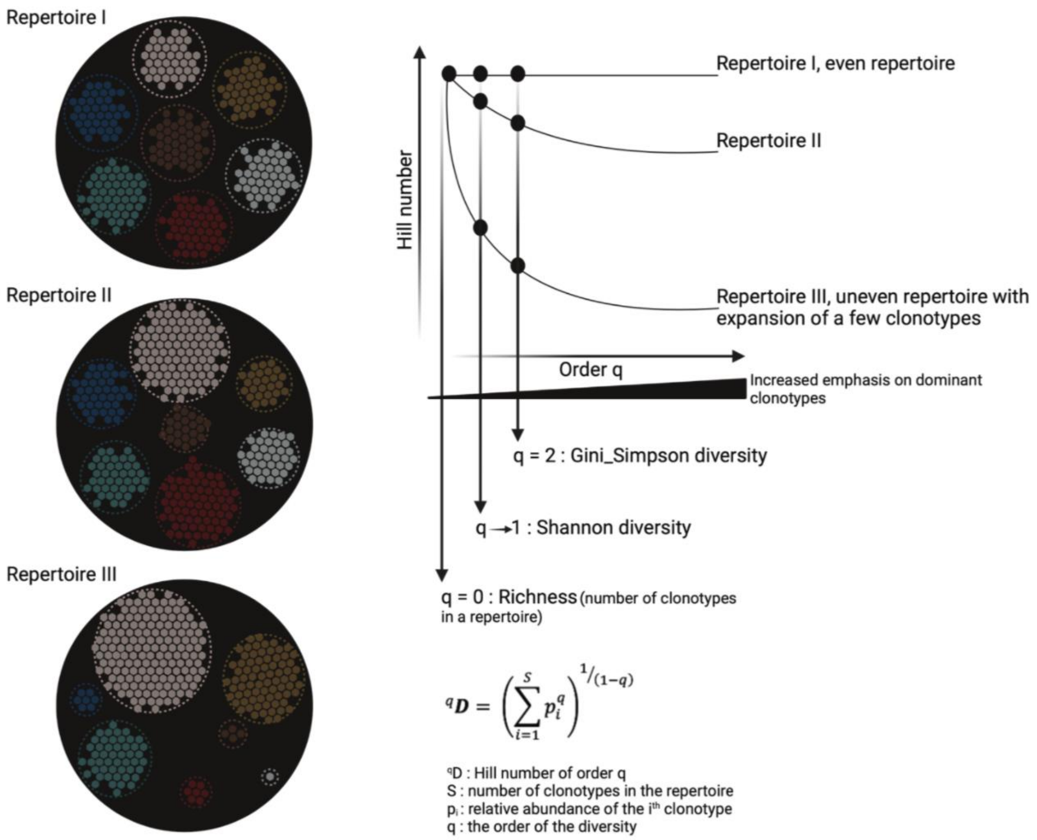

$$^{q}D = \left(\sum_{i=1}^{S} p_i^q\right)^{1/(1-q)}$$

Online Supplemental Figure S4. Waterfall plots showing best response according to RECIST by histology. (A) Arm A. (B) Arm B. (C) Arm C. Dashed horizontal lines at +20% and –30% represent thresholds for progressive disease and partial response, respectively, according to Response Evaluation Criteria In Solid Tumors (V1.1).

A

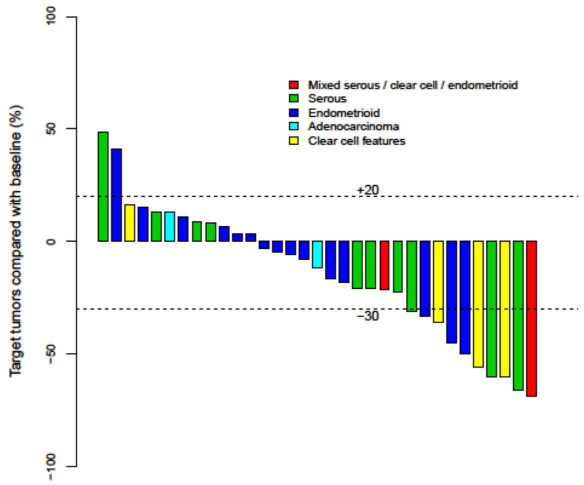

B

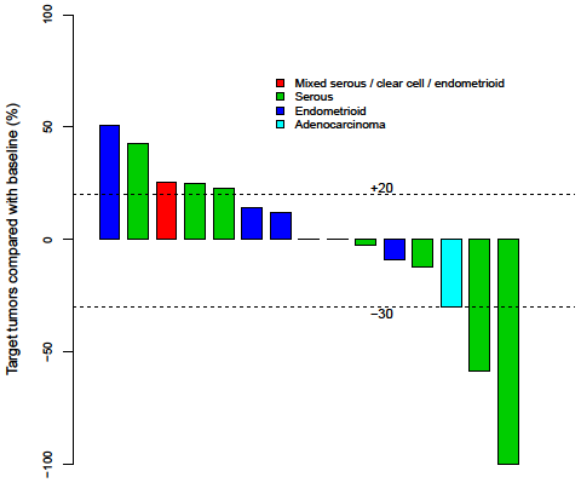
Target tumors compared with baseline (%)
100
50
0
−50
−100
Mixed serous / clear cell / endometrioid
Serous
Endometrioid
Adenocarcinoma
+20
−30

C

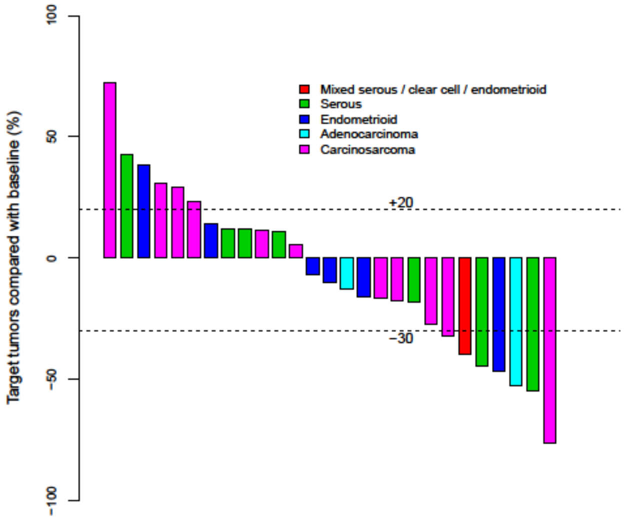
Target tumors compared with baseline (%)
100
50
0
−50
−100
Mixed serous / clear cell / endometrioid
Serous
Endometrioid
Adenocarcinoma
Carcinosarcoma
+20
−30

Online Supplemental Figure S5. Marker expression in select cell clusters (from biopsies). Viably frozen single-cell suspensions from fresh baseline biopsies were stained with a 36-marker cytometry by time of flight panel. CD45+EpCAM- cells from all patients (n=40) were pooled for unsupervised clustering using *phenograph*. (A) Histograms show the signal intensity of CD45RA and CD45RO expression on clusters that are significantly more abundant in Arm A baseline biopsies (red) or more abundant in Arm C baseline biopsies (blue) as shown in figure 4C. (B) Histograms show the signal intensity of select markers in clusters *2* (red), *13* (blue), and *18* (green) CD8+ T cells from baseline biopsies. EpCAM, epithelial cell-adhesion molecule; HLA-DR, human leukocyte antigen – DR isotype; PD-1, programmed cell death-1.

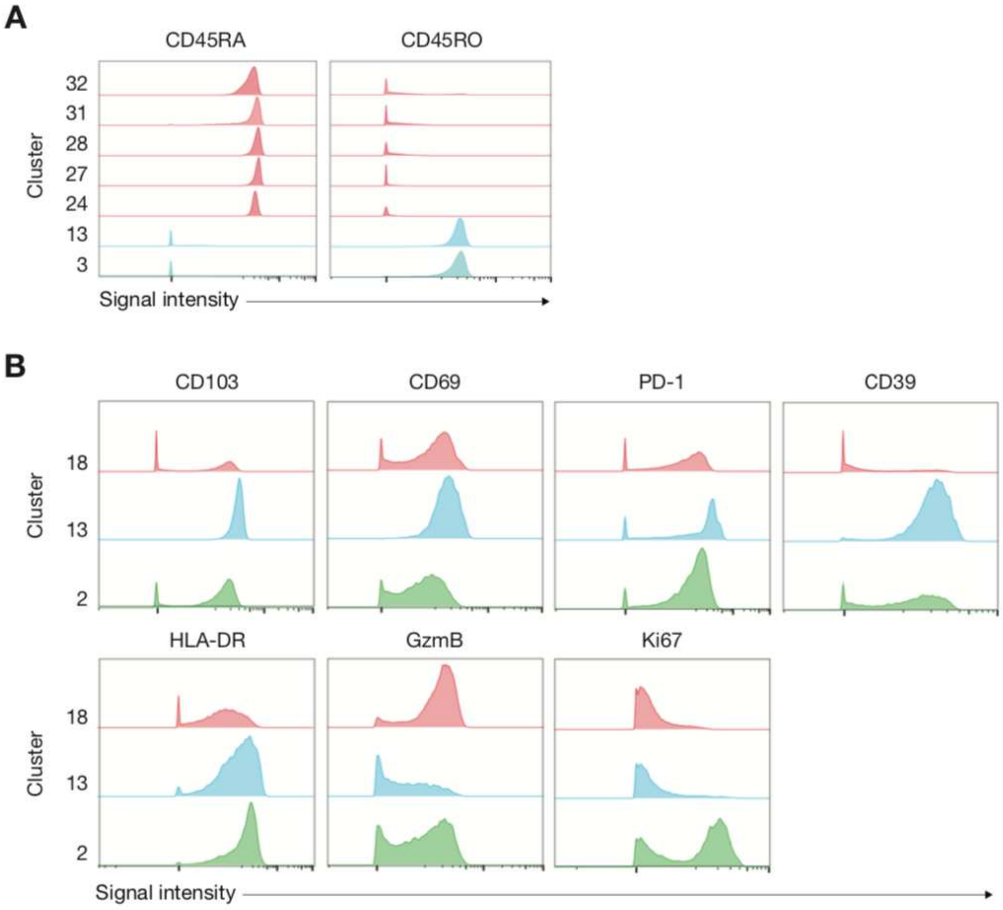
A
CD45RA
CD45RO
Cluster
32
31
28
27
24
13
3
Signal intensity
B
CD103
CD69
PD-1
CD39
Cluster
18
13
2
HLA-DR
GzmB
Ki67
Signal intensity

Online Supplemental Figure S6. Changes in immune cell populations pre- and post-nivolumab monotherapy. (A) Bar graph shows the differential abundance of each immune cell cluster between Arm B before nivolumab (pre-nivo) and paired Arm C after nivolumab (post-nivo) baseline biopsies for Arm B to C crossover patients (n=5). Data are presented as the log fold-change (logFC). LogFC >0 indicates abundant pre-nivo; logFC <0 indicates less abundant pre-nivo. (B) Graphs show the proportion of cells from each *phenograph*-defined cluster pre- and post-nivo among CD45+EpCAM- cells. Patient A who progressed on Arm B but responded on Arm C is highlighted in blue. Patient B who progressed on Arm B but had SD ≥3 months on Arm C is highlighted in yellow. Patients with microsatellite instability-high disease and those with carcinosarcoma were excluded. EpCAM, epithelial cell-adhesion molecule; PD, progressive disease; PR, partial response; SD, stable disease.

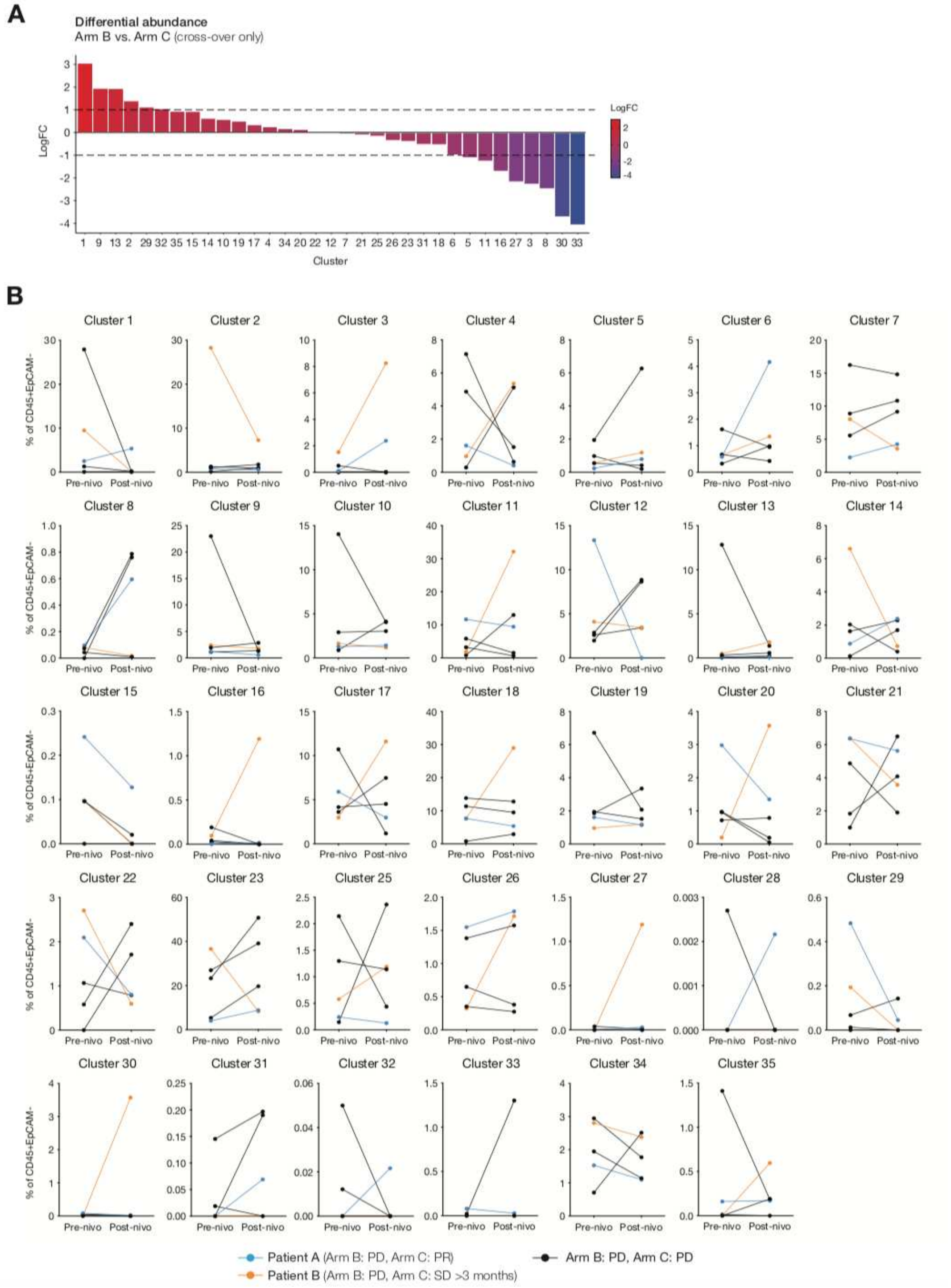
A
Differential abundance
Arm B vs. Arm C (cross-over only)
LogFC
Cluster
B
Cluster 1
Cluster 2
Cluster 3
Cluster 4
Cluster 5
Cluster 6
Cluster 7
Cluster 8
Cluster 9
Cluster 10
Cluster 11
Cluster 12
Cluster 13
Cluster 14
Cluster 15
Cluster 16
Cluster 17
Cluster 18
Cluster 19
Cluster 20
Cluster 21
Cluster 22
Cluster 23
Cluster 25
Cluster 26
Cluster 27
Cluster 28
Cluster 29
Cluster 30
Cluster 31
Cluster 32
Cluster 33
Cluster 34
Cluster 35
% of CD45+EpCAM-
Pre-nivo
Post-nivo
Patient A (Arm B: PD, Arm C: PR)
Patient B (Arm B: PD, Arm C: SD >3 months)
Arm B: PD, Arm C: PD

Online Supplemental Figure S7. *TRGV-TRGJ* gene segment usage. *TRGV-TRGJ* pairing landscapes of peripheral ɣδ T cells collected from three patients at time points T1 (left panel) and T3 (right panel). In each plot the left strata illustrate the V gene segments and the right strata illustrate the J gene segments. *TRGV* and *TRGJ* gene segments are connected via alluvia whose thickness is proportional to the number of clonotypes with the respective V–J pairing in each repertoire. Each V and J gene stratum has a fixed color throughout all panels. Characterization of the *TRG* VJ gene segment usage shifts from T1 to T3 for Patient A (best response: partial response), Patient D (best response: progressive disease), and Patient E (best response: progressive disease) showed that V gene segments associated with a larger number of J gene segments in T3 compared with T1 in the patient who partially responded in Arm C, whereas for two patients who continued to progress in Arm C, V gene segments associated with either the same number or fewer J gene segments in T3 compared with T1. BR, best response; PD, progressive disease; PR, partial response; TCR, T-cell receptor; *TRG*, TCR γ gene.

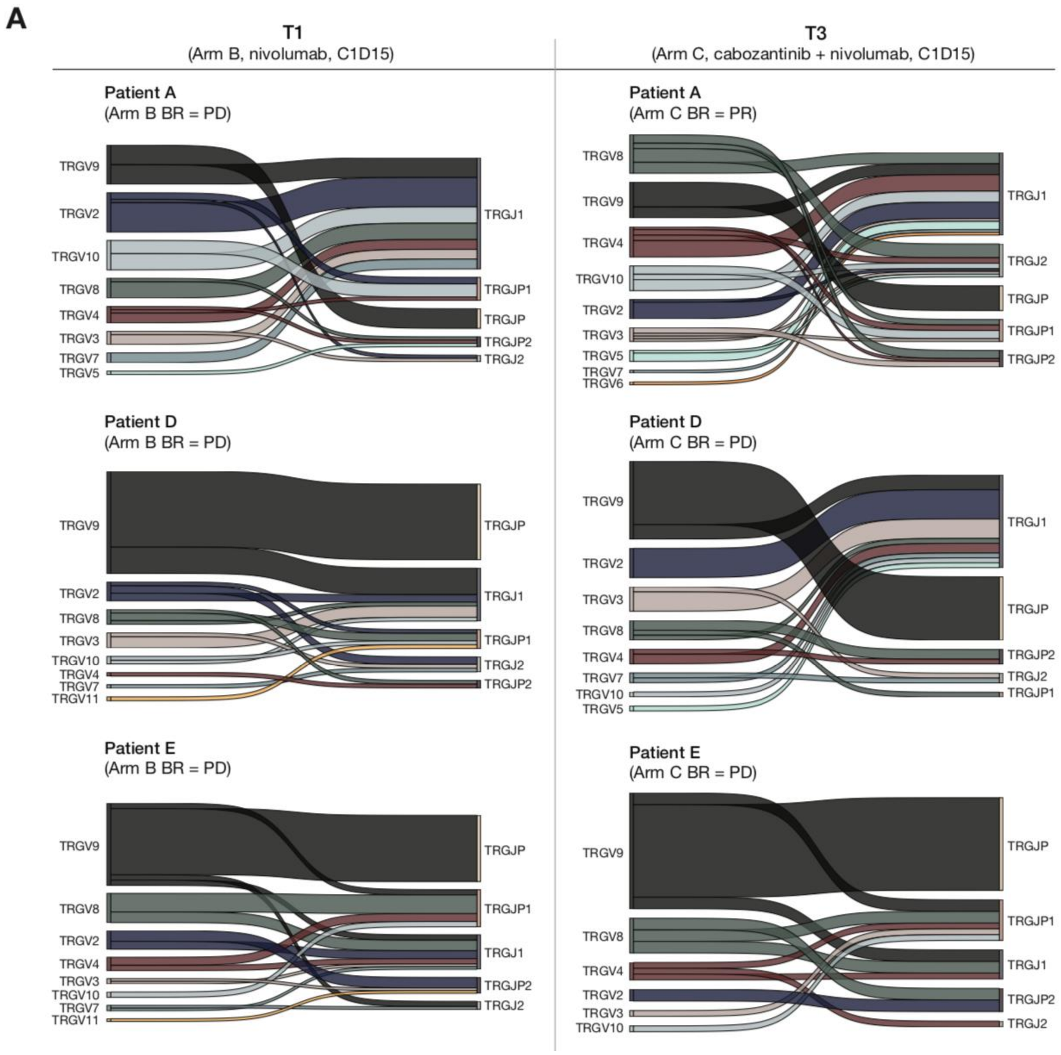
A
T1
(Arm B, nivolumab, C1D15)
T3
(Arm C, cabozantinib + nivolumab, C1D15)
Patient A
(Arm B BR = PD)
Patient A
(Arm C BR = PR)
Patient D
(Arm B BR = PD)
Patient D
(Arm C BR = PD)
Patient E
(Arm B BR = PD)
Patient E
(Arm C BR = PD)
TRGV9
TRGV2
TRGV10
TRGV8
TRGV4
TRGV3
TRGV7
TRGV5
TRGV6
TRGV11
TRGJ1
TRGJP1
TRGJP
TRGJP2
TRGJ2
